# Implementation of a Triage Protocol Outside the Hospital Setting for Timely Referral During the COVID-19 Second Wave in Chennai, India

**DOI:** 10.2196/42798

**Published:** 2023-12-18

**Authors:** Alby John, Jagadeesan M, Polani Rubeshkumar, Parasuraman Ganeshkumar, Hemalatha Masanam Sriramulu, Manish Narnaware, Gagandeep Singh Bedi, Prabhdeep Kaur

**Affiliations:** 1 Greater Chennai Corporation Government of Tamil Nadu Chennai India; 2 Indian Council of Medical Research-National Institute of Epidemiology Chennai India

**Keywords:** COVID-19, triage, low- and middle-income countries, LMIC, India, pulse oximeter, implementation, health care system, self-management, patient care, community health, low income, health disparity, low-resource setting

## Abstract

India experienced a surge in COVID-19 cases during the second wave in the period of April-June 2021. A rapid rise in cases posed challenges to triaging patients in hospital settings. Chennai, the fourth largest metropolitan city in India with an 8 million population, reported 7564 COVID-19 cases on May 12, 2021, nearly 3 times higher than the number of cases in the peak of COVID-19 in 2020. A sudden surge of cases overwhelmed the health system. We had established standalone triage centers outside the hospitals in the first wave, which catered to up to 2500 patients per day. In addition, we implemented a home-based triage protocol from May 26, 2021, to evaluate patients with COVID-19 who were aged ≤45 years without comorbidities. Among the 27,816 reported cases between May 26 and June 24, 2021, a total of 16,022 (57.6%) were aged ≤45 years without comorbidities. The field teams triaged 15,334 (55.1%), and 10,917 (39.2%) patients were evaluated at triage centers. Among 27,816 cases, 19,219 (69.1%) were advised to self-isolate at home, 3290 (11.8%) were admitted to COVID-19 care centers, and 1714 (6.2%) were admitted to hospitals. Only 3513 (12.7%) patients opted for the facility of their choice. We implemented a scalable triage strategy covering nearly 90% of the patients in a large metropolitan city during the COVID-19 surge. The process enabled early referral of high-risk patients and ensured evidence-informed treatment. We believe that the out-of-hospital triage strategy can be rapidly implemented in low-resource settings.

## Introduction

India experienced a surge of cases and deaths in the second wave of COVID-19 in 2021. The highest number of daily cases was 0.4 million on May 6, 2021, which was 4 times the highest number of reported cases in the first wave [1]. The rapid rise in cases overwhelmed the health system. There was a surge in hospital admissions, and many patients required either an oxygen bed or intensive care units during the peak [2]. The sudden surge in the number of cases was attributed to the highly infectious Delta mutant variant of COVID-19 (B.1.617 lineage) and lack of compliance with COVID-19–appropriate behaviors [3]. Tamil Nadu, one of the Southern States in India, had nearly 1.2 million cases and 17,855 deaths between May 01, 2021, and June 24, 2021 [4].

## Local Setting

With a population of 8 million, Chennai city is the capital of Tamil Nadu state in India. Greater Chennai Corporation (GCC) is the administrative authority of Chennai, with 15 administrative zones and 200 divisions. Chennai reported a maximum of 2358 cases on June 30, 2020, during the first wave [1]. During the second wave, the highest reported cases were 7564 on May 12, 2021, nearly thrice as high as the number of cases in the peak of the first wave [1].

Having no preplanned pandemic response, GCC adopted a community-centered, patient-friendly strategy devised by a multidisciplinary team of public health experts to combat the first wave of the COVID-19 pandemic [5]. The key strategies included surveillance, testing, contact tracing, triage centers, facility-based isolation, supervised home isolation, and quarantine [5]. These strategies were implemented at once in the early phases of the first wave of the pandemic [5]. Establishing triage centers on an ad hoc basis outside the hospital settings for early identification of severe COVID-19 was one of the strategies. GCC established 12 triage centers across all parts of Chennai in 2 weeks in the first wave (April-June 2020), which remained functional until the end of the second wave of the pandemic [5]. The triage centers were conveniently located in public buildings, such as educational institutions, community halls, and stadiums for easy access. Information about these triage centers was communicated through social media. All the services were free of charge in public sector facilities and under the Government Comprehensive Health Insurance scheme in private health facilities for eligible beneficiaries.

There were challenges in implementing these strategies, especially due to the scarce workforce. Tamil Nadu state has a well-structured, well-staffed state-level public health department with a highly trained workforce catering predominantly to the rural population. This workforce was mobilized to support various managerial and field-level activities, such as surveillance, contact tracing, sample collection, clinical care, and strategic planning in Chennai. These mobilized, trained workforces joined the health care team of GCC and implemented the strategies. Health care workers were used for technical activities, such as testing and treatment. Additionally, a non–health care workforce and trained community volunteers were used for strategic planning and logistics. GCC used these health care and non–health care workforces for the functions of triage centers. The combination of public health strategies, test-trace-isolate, and appropriately timed restrictions helped control the first wave without burdening the health system [5].

**Figure 1:**
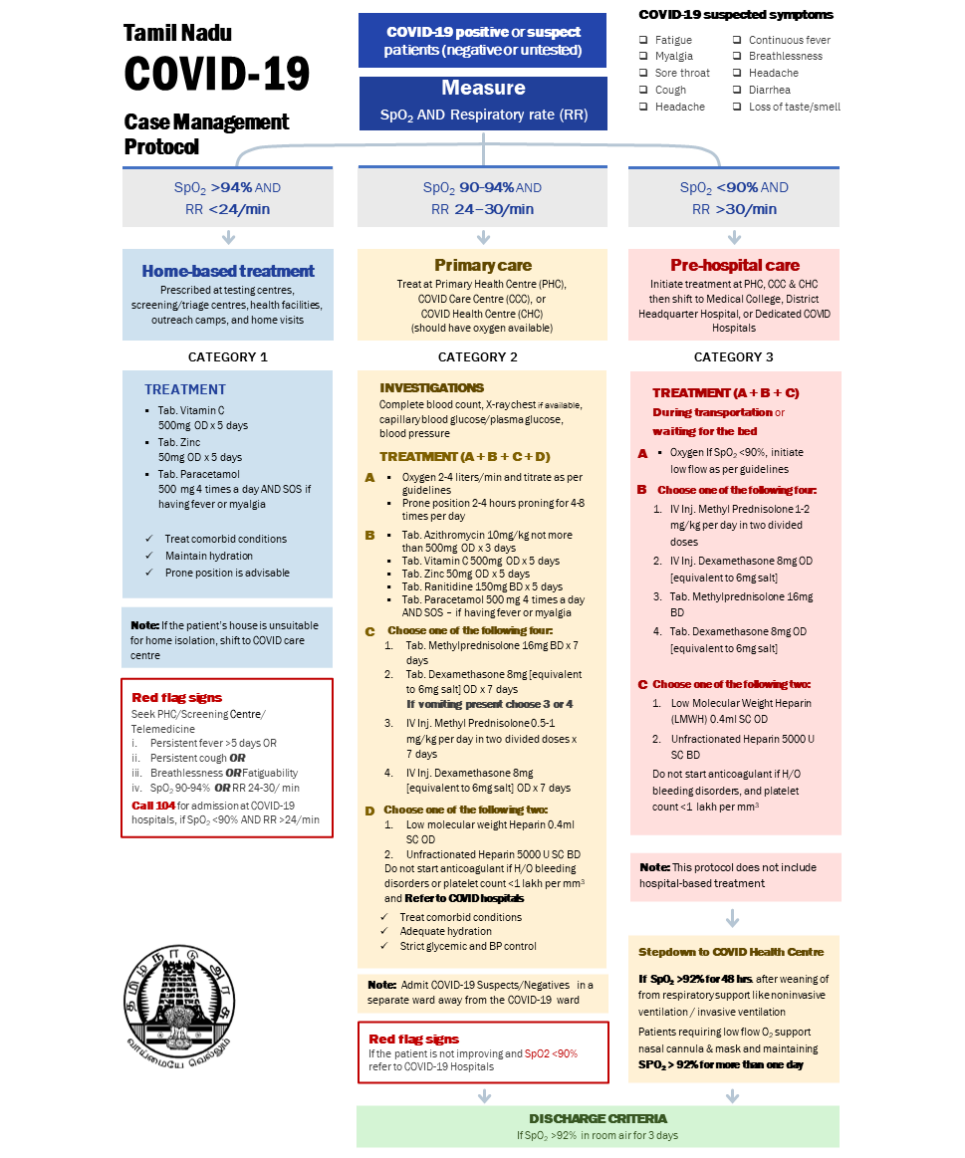
COVID-19 triage protocol, Tamil Nadu, India. BD: twice daily; BP: blood pressure; H/O: history of; Inj: injection; O_2_: oxygen; OD: once daily; SC: subcutaneous; SOS: taken as required; SpO_2_: oxygen saturation; Tab: tablet.

The sudden surge of cases overwhelmed the health system. More than 90% of oxygen beds were occupied on May 24, 2021 [4]. The 12 triage centers could evaluate up to 2500 cases per day, which was inadequate during the second wave. Inability to assess patients within 24 hours after diagnosis led to panic among patients. They either rushed to hospitals or called helplines for evaluation, treatment, and hospital beds. The Government of Tamil Nadu introduced a new triage protocol to evaluate patients at home or in field-based settings (Figure 1) [6]. GCC developed a patient-centric, field-based strategy to assess confirmed cases of COVID-19 at home or in settings close to their homes according to the protocol. This paper describes the feasibility, challenges, and lessons learned from a patient-centric, outside-the-hospital triage strategy.

## Description of the Triaging Intervention

On May 26, 2021, we implemented an evidence-informed triage protocol for COVID-19 cases at a field level. Beside the 12 triage centers established in the first wave of the pandemic, we formed field triage teams with a repurposed workforce of 800 paramedics and 200 doctors. Each field triage team could screen 80-100 cases per day; altogether, the 200 field triage teams had the capacity to screen an additional 20,000 patients per day. Each field triage team had 4 paramedics and 1 doctor and catered to one of the 200 divisions.

Patients were tested first in one of the facilities with real-time reverse transcription polymerase chain reaction testing. These facilities included mobile testing teams, public sector walk-in testing centers, private sector labs, and hospitals. Irrespective of the testing center, all results were uploaded to an integrated web-based data management portal. GCC emergency operation center collected the line list, stratified them by divisions and sent them to the respective triage teams. Each team was supplied with a line list of COVID-19–positive cases. A member of the field triage team contacted the allotted cases, informed the time of the home visit, and screened the patient at the doorstep. Each team member assessed a maximum of 30 COVID-19 cases at the doorstep. Every team member had a thermal scanner, pulse oximeter, sphygmomanometer, glucometer, personal protective equipment, hand sanitizer, and vehicle for logistics. The equipment were procured through pandemic relief funds and donations. The protocol enabled the paramedic to do the initial evaluation and make decisions regarding home isolation or hospitalization after consultation with a doctor.

The triage team referred patients ≥45 years of age and patients <45 years of age with comorbidities to triage centers directly. The team visited patients ≤45 years without any comorbidities. A paramedic asked for the symptoms and comorbidities and measured oxygen saturation with the pulse oximeter. If the patient had oxygen saturation (SpO_2_) <94% or high-grade fever >38.8 °C, they were referred to triage centers for detailed evaluation.

The standalone triage centers outside the hospitals had 4 doctors and 8 paramedics. The centers were equipped with pulse oximeters, thermal scanners, sphygmomanometers, a chest x-ray unit, and a cell counter. The doctors referred patients with mild to moderate illness or those with a lack of adequate facilities for home isolation to facility-based isolation units known as “COVID Care Centers” (CCC). Patients with COVID-19 who had severe infections were referred to the hospital.

The criteria for home isolation for patients triaged at home or standalone triage centers were mild symptoms and SpO_2_ >94%. Other criteria for home isolation were the availability of a separate room with an attached bathroom or toilet and a caregiver. Patients were given a home isolation kit with medications, namely paracetamol, vitamin C, and zinc tablets [6]. Doctors in the telemedicine center followed up with patients in home isolation for 10 days. If they reported red flag signs, they were transferred to the hospital by special ambulances.

## Ethical Considerations

This study has been approved by the institutional human ethics committee of Indian Council of Medical Research-National Institute of Epidemiology, Chennai, India (NIE/IHEC/202004-07). Informed consent was not obtained because the survey team did not interview any human participants. We did not handle any information containing personal identifiers. No monetary or other benefits were given to the participants of the study.

## Results

**Figure 2:**
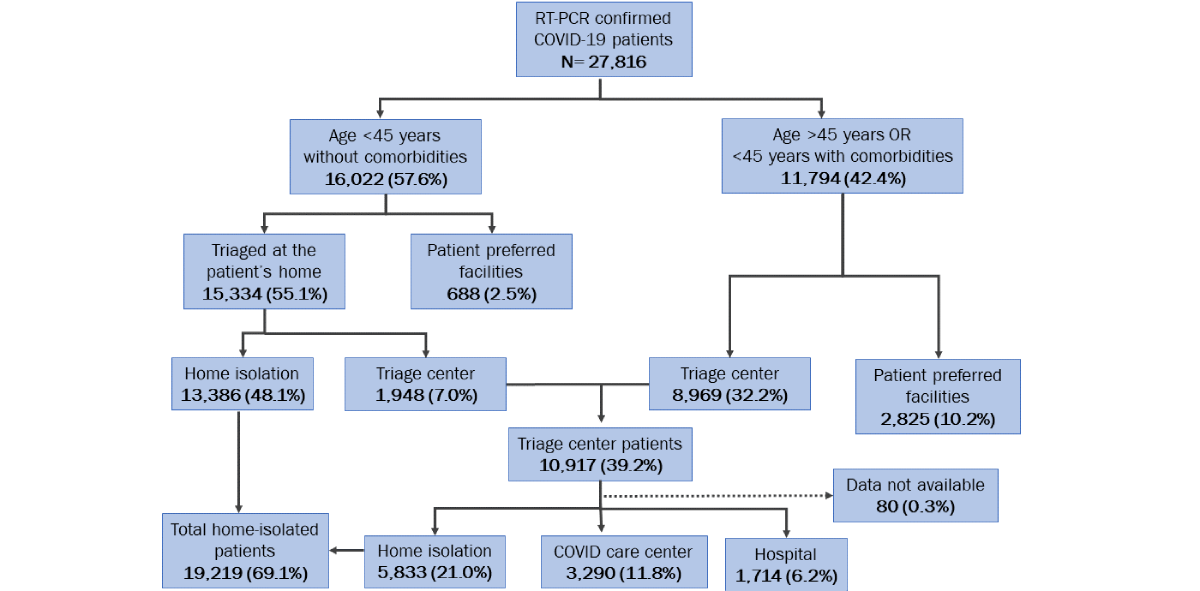
Outcomes of the out-of-hospital triage of the COVID-19 cases in Chennai, Tamil Nadu, India during May-June 2021. RT-PCR: real-time reverse transcription polymerase chain reaction.

We analyzed the triaging data from May 26, 2021, to June 24, 2021. Overall, Chennai reported 27,816 cases during this period. Among the reported cases, 16,022 (57.6%) were aged <45 years with no comorbidities, hence eligible for triaging at home. The field teams triaged 15,334 (55%), and the rest opted for evaluation in the facility of their choice. Among those who underwent triage at home, 13,386 (48%) were recommended home isolation, and 1948 (7%) were referred to triage centers (Figure 2).

Of the 27,816 cases, 11,794 (42.4%) were ≥45 years or <45 years with comorbidities. Nearly one-third of the patients (n=8969) were directly evaluated at standalone triage centers, in addition to 1948 referred after triaging at home. Only 2825 (10%) sought treatment at facilities of their choice (Figure 2).

Overall, the doctors advised home isolation for 19,219 (69.1%) of the patients in this period. All the patients in home isolation were followed for 10 days through telemedicine and home visits, if required. Among those in home isolation, we identified 271 (1.4%) with SpO_2_ <94% and referred them to secondary or tertiary centers for treatment.

Nearly one in 10 (11.8%) patients were admitted to CCCs, which catered to patients who did not have facilities for home isolation or had symptoms requiring monitoring of SpO_2_ in the range of 90%-94%. According to the treatment protocol, the CCC was equipped with oxygen concentrators and medications. Only 1714 (6%) patients required hospitalization (Figure 2).

### Lessons Learned

We executed a field-based triage strategy that enabled access to timely evaluation for nearly half of the cases at home and another 40% in the nearest standalone triage center during the surge of the COVID-19 cases. The strategy was feasible and rapidly scalable in a large metropolitan city, such as Chennai (Textbox 1). The process enabled early identification and referral of patients with moderate to severe illness, including silent hypoxia. The protocol was pragmatic and based on the oxygen saturation levels using pulse oximeters, an objective tool for clinical evaluation [7]. Globally, the experts recommend pulse oximetry as one of the best triaging tools [7]. Timely procurement and distribution of pulse oximeters to all triage teams played a significant role in the successful implementation of the protocol. This approach ensured that ambulances and scarce oxygen beds were allocated to patients who needed it the most, irrespective of their affluence or socioeconomic status.

Ensuring access to health care facilities is essential and critical in preventing deaths and panic among patients [8]. The process reduced crowding in the hospital clinics. Low- and middle-income countries, like India, often experience a shortage of workforce during the routine functioning of health care, and such shortages may worsen during times of surge [9]. This approach prevented the sudden influx of all patients to the hospitals, and the precious human resources were well used for COVID-19 management. On August 6, 2021, the protocol was modified, and the admission criteria were relaxed after the number of cases decreased. The patients were referred to secondary or tertiary care institutions if they required hospitalization [10]. Tamil Nadu state adopted Government of India COVID-19 treatment guidelines. The Government of India has modified the treatment guidelines several times based on the best available evidence, and the latest guidelines were published on January 5, 2023 [11].

The community acceptance of the intervention was high. The good handling of the first wave of the pandemic by GCC gained the trust of the community [5,12]. Besides, the high cost of the private health care system was concerning [13]. There were reports of overuse of computed tomography scans and irrational medications in the second wave in India [14]. On the other hand, the services in the public health system were of good quality and easily accessible at no cost. These reasons could have motivated the community to use the services in the public health system. The protocol included only evidence-informed evaluation and treatment, hence minimized the use of irrational diagnostics and drugs. The early isolation of cases reduced the disease spread in the community. Globally, the telemedicine approach for the assessment, evaluation, and follow-up of patients with COVID-19 is well accepted [15]. This protocol incorporated telemedicine-based follow-up of patients until recovery for identifying red flags and timely referrals.

One limitation was lack of data regarding 3513/27,816 (12.7%) of the patients who opted for facilities of their choice. We might have underestimated the overall hospitalizations due to a lack of information about this group. Similarly, we could not analyze the data of 80 (0.3%) patients evaluated in triage centers due to reporting errors.

The strength of our approach was that we generated real-time evidence of a triaging protocol for patients in or closer to their homes and outside the hospital settings.

The strategy is replicable and can be used in low-resource settings during the COVID-19 surge or similar outbreaks. The field-based approach reduces spread, ensures timely referral, saves lives, and ensures appropriate use of scarce resources.

**The feasibility of triaging at a field level for early referral of high-risk patients with COVID-19 in a large metropolitan city.**

**Key findings**

- Measurement of oxygen saturation through pulse oximetry is a simple objective tool to triage COVID-19 cases in low-resource settings and for early identification of hypoxia.
- Triaging patients with COVID-19 outside hospitals was feasible and rapidly scalable in a large metropolitan city.
- Field triaging of COVID-19 was patient-friendly and well accepted by the community; it reduced panic among the public and crowding in the hospitals.

**Key implications**

- Field triaging is a feasible strategy to identify and refer high-risk patients in low-resource settings
- Pulse oximeter is a simple tool to quantitatively triage patients with COVID-19 at a field-based setting
- Field-triaging strategy could reduce additional burden on health facilities

## Abbreviations

CCC: COVID Care Center
GCC: Greater Chennai Corporation
SpO_2_: oxygen saturation

## Data availability

The data sets generated and analyzed during this study are not publicly available, as they are considered classified public health information, but they are available from the corresponding author upon reasonable request.
